# Factors Associated with Malnutrition and Its Impact on Postoperative Outcomes in Older Patients

**DOI:** 10.3390/jcm10122550

**Published:** 2021-06-09

**Authors:** Maria Venianaki, Alexandros Andreou, Taxiarchis Konstantinos Nikolouzakis, Emmanuel Chrysos, George Chalkiadakis, Konstantinos Lasithiotakis

**Affiliations:** 1 Department of General Surgery, University General Hospital of Heraklion, 71110 Heraklion, Crete, Greece; mb12.med@gmail.com (M.V.); alexandrosand@hotmail.com (A.A.); medp2011836@med.uoc.gr (T.K.N.); chrysose@uoc.gr (E.C.); kwstaslasith@yahoo.gr (G.C.); 2 Department of Anatomy, Medical School, University of Crete, 71110 Heraklion, Crete, Greece

**Keywords:** geriatric surgery, general surgery, malnutrition, malnutrition universal screening tool

## Abstract

Malnutrition is associated with dismal treatment outcomes in older patients but its impact in geriatric surgery has not been studied extensively. Herein, we report the prevalence of malnutrition risk, its risk factors and its association with postoperative outcomes in older patients undergoing operations of general surgery. This is a retrospective analysis of a prospectively maintained database including patients older than 65 years who were to undergo general surgery operations between 2012 and 2017. The Malnutrition Universal Screening Tool (MUST) was used for nutritional risk. Demographics, socioeconomic data, site and magnitude of the operation, various measures of comorbidity and functional dependence as well as postoperative complications based on Clavien–Dindo classification and length of stay were recorded. There were 501 patients. A total of 28.6% of them were at intermediate malnutrition risk (MUST = 1) and 14.6% were at high malnutrition risk (MUST ≥ 2). Variables independently associated with malnutrition risk (MUST ≥ 1) were smoking (Odds Ratio, OR:1.6, *p* = 0.041), upper gastrointestinal (GI) tract surgery (OR:20.4, *p* < 0.001), hepatobiliary-pancreatic surgery (OR:3.7, *p* = 0.001), lower GI surgery (OR:5.2, *p* < 0.001) and American Society of Anesthesiologists (ASA) class III/IV (OR:2.8, *p* = 0.001). In the multiple regression analysis adjusted for several confounding variables, the MUST score was significantly associated with postoperative death (OR:9.1, *p* = 0.047 for MUST = 1 and OR:11.9, *p* = 0.035 for MUST score ≥ 2) and postoperative hospital stay (adjusted incidence rate ratio, 1.3, *p* = 0.041 for MUST = 1 and 1.7, *p* < 0.001 for MUST ≥ 2). Malnutrition risk was highly prevalent in this sample, particularly in patients with operations of the gastrointestinal tract, in patients with poor physical status and it was associated with postoperative mortality and length of stay.

## 1. Introduction

The number of geriatric surgical patients is increasing because of the aging of the population and advances in medicine, which allow even major operations to be performed safely in older patients. However, increasing age is associated with dismal surgical outcomes and this is due to reduced functional reserves while social and economic deprivation might also play a role. Malnutrition is a pivotal factor in surgery as it is frequently a cause and a result of postoperative complications. Moreover, malnutrition is quite prevalent in older patients in the hospital reaching 50% in Western European countries [1,2]. In general surgery, the prevalence of malnutrition risk ranges between 20 and 50% [3,4]. More than 80% of the patients undergoing major hepatobiliary/pancreatic (HPB) and bowel surgery might be at risk of malnutrition or malnourished and this condition has been invariably associated with dismal surgical outcomes [5,6]. Moreover, malnutrition is considered a potentially modifiable risk factor and a pillar of modern prehabilitation programs with the aim to ameliorate postoperative morbidity [7,8,9,10]. Despite that fact, the prevalence of malnutrition in geriatric surgery patients and its impact on postoperative outcomes has not received much research attention so far. The aim of this study is to identify factors associated with the risk of malnutrition in older patients undergoing operations in the specialty of General Surgery and to determine the association of malnutrition with postoperative complications and length of stay.

## 2. Materials and Methods

### 2.1. Study Population

Between 2012 and 2017, patients 65 years and older, who were to undergo operations within the specialty of General Surgery were included in this study. These included abdominal wall hernia repairs, operations of the gastrointestinal tract including liver and pancreas, operations of soft tissue and of endocrine glands. Acute and emergency operations of this type were included. There were no operations for abdominal trauma in this sample. There were no orthopedic operations, no neurosurgical operations or bariatric operations in this sample. Patients who were not able to undergo nutritional assessment (i.e., due to poor physical or mental condition) and/or provide written consent were excluded. Written informed consent was signed by all patients and the study protocol was approved by the Institutional Scientific and Ethical Committee of the University Hospital of Heraklion. All consenting patients underwent physical examination and an interview by a senior surgical trainee up to 24–48 h prior to an elective operation and immediately prior to an emergency operation. During the interview all the assessments and tests mentioned below were carried out. In patients with cognitive impairment, necessary information was gathered or was confirmed by their closest relative or caregiver.

### 2.2. Perioperative Data

For nutritional assessment, the MUST tool was used before the operation. MUST was developed by the British Association for Parenteral and Enteral Nutrition (BAPEN) in order to identify adults at risk of malnutrition [11]. A score of 0, 1 and ≥2 denotes low, medium and high risk for malnutrition, respectively. Self-maintaining and instrumental activities were assessed using the Katz basic activities of daily living index (ADL) which includes 6 items that assess basic self-care activities such as bathing, dressing, clothing, toilet, feeding, transfer and continence [12]. A score of 0–2 denotes a dependent patient, 3–4 an intermediate and 5–6 an independent patient. Comorbidity was assessed using the Charlson comorbidity index (CCI) [13]. The American Society of Anesthesiologists (ASA) classification is a physical status classification system which consists of 5 categories of increasing severity [14]. Very briefly, ASA class I denotes a completely healthy fit patient. ASA II and III denote a patient with mild systemic and severe systemic disease that is not incapacitating, respectively. ASA IV refers to a patient with an incapacitating disease that is a constant threat to life and class V a moribund patient. Indicators of financial status, educational level, and measures of social reserves (such as living with partner, presence of kids) were also recorded. History of smoking was defined as reported smoking of cigarettes in the year before admission for surgery. Alcohol use was defined as the consumption of >7 standard drinks per week or >3 drinks on any day. The history of falls was defined according to the World Health Organization as an event which results in a person coming to rest inadvertently on the ground or floor or other lower level. The diagnosis of dementia was registered if the patient has previously had a formal diagnosis by a specialist (i.e., neurologist, psychiatrist, general practitioner or internal medicine specialist). The current diagnosis of cancer was also registered. The magnitude of the operations was assessed using the Physiological and Operative Severity Score for the enumeration of Mortality (POSSUM) categories (minor, intermediate, major, major plus) and the site of operation was grouped in 6 categories (hernia, upper GI, hepatobiliary/pancreatic, cholecystectomy, lower GI, soft tissue/other) [15].

Postoperative complications and length of stay were prospectively registered in the database. Complications were defined as any deviation from the normal postoperative course and were classified into 4 severity grades according to the definitions of Clavien et al. [16]. Grade 1 included minor risk events not requiring therapy. Grade 2 complications were defined as potentially life-threatening complications with the need of intervention or a hospital stay longer than twice the median hospitalization for the same procedure. Grade 3 complications were defined as complications leading to lasting disability or organ resection. A grade 4 complication indicated death of a patient due to a complication. The study ended for each patient at discharge and there was no follow-up of the patients thereafter. No complications of deaths were recorded after the discharge of the patients from the hospital.

### 2.3. Statistical Analysis

Categorical variables were presented as numbers (percentage) and continuous variables are presented as mean (standard deviation, (SD)) if they were normally distributed or as median ((IQR), interquartile range) if they did not follow the normal distribution.

Normal distribution was tested with the use of Q–Q plots and the Kolmogorov–Smirnoff test. Associations between categorical variables were examined using Pearson’s Chi square test. For the comparison of distribution of continuous variables parametric (*t*-test) or non-parametric tests (Mann–Whitney U test) were used. Relative risks were estimated using exposure odds ratios (ORs) and the corresponding 95% confidence intervals (CIs) from cross tabulation. A multiple logistic regression analysis was performed to determine independent factors associated with the prevalence of malnutrition, and the occurrence of postoperative complications. Covariates in each multiple regression model were independently selected if their *p* value on univariable analysis for that particular outcome was 0.05 or less. As postoperative length of stay (LOS) was not normally distributed, negative binomial regression analysis was performed to determine associated factors [17]. All *p* values were two-sided and the significance level was chosen to be 0.05. All calculations were performed with the Statistical Package for Social Sciences (SPSS) ver. 26.0 (SPSS Inc., Chicago, IL, USA).

## 3. Results

A total of 501 patients were included in the final analysis. The median (IQR) age of the population was 74 (10) years. The demographics and perioperative variables are shown in Table 1.

The overall prevalence of intermediate malnutrition risk in this sample (score = 1) was 28.3%. A total of 14.6% of the patients were at high malnutrition risk (score ≥ 2). In the univariate analysis, factors associated significantly with malnutrition risk were financial status, smoking, Katz-ADL categories, the history of falls, Charlson’s comorbidity index, diagnosis of cancer, diagnosis of dementia, POSSUM category of operation, ASA class and the site of the operation (Table 1). The results of the detailed univariate analysis using three categories of malnutrition risk (MUST score, 0/1/ ≥ 2) are similar and are presented in the Supplementary Table S1. Patients undergoing major or major plus operations, operations in the upper GI/lower GI and HPB site and patients classified as ASA III/IV were more often at malnutrition risk. In the multiple regression analysis, variables significantly associated with malnutrition risk (MUST score ≥ 1) were smoking (OR:1.6), operation in the upper GI (OR:20.4), HPB (OR:3.7), lower GI (OR: 5.2) and ASA class II (OR:1.7) or III/IV (2.8). The detailed results of the multiple regression analysis are presented in Table 2.

The distribution of preoperative variables according to grades of postoperative complications are presented in detail in Table 3.

In the univariate analysis the MUST score was significantly associated with the occurrence of any postoperative complication (*p* = 0.001) and postoperative death (*p* = 0.001) but the association did not reach statistical significance for the prevalence of serious postoperative complications (Clavien–Dindo ≥ 3) (*p* = 0.079). The respective ORs from crosstabulation are presented in Table 4 along with the adjusted OR (AOR) derived from multiple logistic regression analysis. The detailed results of the multivariate analysis are presented in the Supplementary Tables S2–S4.

In the multiple regression analysis, the association of the MUST score with postoperative complications retained its statistical significance for postoperative death (AOR (95% CI: 9.1(1.1–80.3), *p* = 0.047) for MUST score = 1 and 11.9 (1.2–121), *p* = 0.035 for MUST score ≥ 2). The median (IQR) length of postoperative stay was significantly longer in patients at medium (MUST score = 1) or high (MUST score ≥ 2) risk for malnutrition compared with patients at low risk of malnutrition (MUST score = 0) (*p* < 0.001) (Table 4). Other factors linked with longer postoperative stay were the site of the operation (*p* < 0.001), Charlson’s index (*p* < 0.001) and ASA class (*p* < 0.001), polypharmacy (*p* = 0.034) and diagnosis of cancer (*p* < 0.001) (data not presented). In the multiple regression analysis, patients with medium or high nutritional risk had a longer postoperative stay (adjusted incidence rate ratio (95% CI), 1.3 (1.01–1.6), *p* = 0.041 for MUST = 1 and 1.7 (1.5–2.0), *p* < 0.001 for MUST ≥ 2). The detailed results of the multiple negative binomial regression analysis are presented in the Supplementary Table S5.

## 4. Discussion

This study has assessed various clinical and social variables as predictors of malnutrition risk in older patients undergoing general surgery. The history of smoking, bowel or HPB surgery and high ASA class were identified as the independent predictors of malnutrition risk in this sample. Patients at medium or high risk for malnutrition more often experienced postoperative complications and significantly longer postoperative stays. The impact of malnutrition risk remained for postoperative stay and postoperative death after adjusting for several confounding factors such as the site of surgery, the magnitude of the operation, comorbidities, functional independence, diagnosis of cancer etc. These results point out the significance of malnutrition in general geriatric surgery patients and provide rationale for nutritional assessment to those who are at greater risk for dismal postoperative outcomes.

The MUST screening tool, which is a validated score questionnaire, was used to identify patients at risk of malnutrition in this study. Currently there is no consensus regarding the best screening method for the assessment of malnutrition in surgical patients and this is particularly true for geriatric surgical patients [18]. There is however, a range of tools such as the malnutrition screening tool (MST), the nutrition risk index (NRI), the subjective global assessment (SGA), the mini nutritional assessment short form (MNA-SF) and the nutritional risk screening (NRS-2002). Systematic reviews do not identify significant differences between tools within studies comparing different tools on the same population. Moreover the results between comparative studies have been inconsistent [19]. MUST was developed by a multidisciplinary group of health care professionals based on information of patient groups in medical and surgical wards, in the older and in the community setting. The tool has correlational validity since it shows good to excellent agreement with many other tools and with a dietitian’s assessment of malnutrition risk. Moreover it has predictive validity in patients undergoing various types of surgery in terms of postoperative complications and length of stay [6,19,20,21]. The prevalence of malnutrition in the preoperative setting of gastrointestinal operations has been reported to range from 33% to 88% depending on the population and the screening tools [6,21,22,23,24]. A small number of studies have focused on older surgical patients who are inherently susceptible to malnutrition due to several factors [1,5,9,20]. Some of them are poor dentition, malabsorption of nutrients, impaired swallowing, consumption of drugs that alter the taste and the appetite as well as socioeconomic factors which limit the access to high quality food [25,26]. In a multicenter Belgian study, 66% of patients over the age of 70 who were admitted in to hospital in order to undergo major elective abdominal surgery were at high risk of malnutrition using the NRS-2002 tool [1]. A retrospective study from Singapore reported a lower prevalence of malnutrition risk defined as MUST ≥ 1 of 11.9% among 1033 older patients admitted for various operations such as general surgery/gynecology, orthopedics and urology. Notably the risk was highest among patients undergoing general surgery/gynecology surgery at 16.3% [20]. Kim et al. using the MNA tool, reported a rate of malnutrition risk/malnourished reaching 88% among older patients undergoing pancreatectomy for periampullary neoplasms [5]. With the use of Geriatric Malnutrition Risk Index 55% of older patients were found to be at high risk for malnutrition [9]. In the latter study a cut off of Geriatric Nutrition Risk Index (GNRI) >92 was used to identify patients at high risk for malnutrition because that was the mean value of the sample. These results are difficult to interpret and compare because of different methods used to assess malnutrition. Thus, it seems imperative that future large-scale comparative studies identify the most suitable screening tool for surgery. In this study, the prevalence of medium/high risk of malnutrition was 45% which is within the wide range of values reported in the literature for geriatric surgery patients (estimated between 20% and 50%) [3,4]. Moreover, it represents the real burden of malnutrition in the older generation in a general surgery department because the sample was not restricted to specific sites or diseases nor were the results of general surgery mixed with the results other disciplines.

This study found an association of malnutrition risk in the preoperative setting with smoking, site of surgery and higher ASA physical status. These risk factors have also been reported in the literature along with loss of appetite, loss of interest in life, dementia, dependence in activities of daily living, functional decline, comorbidity alcohol abuse, chewing and swallowing problems, absence of partner, etc. [20,27]. Smoking has been studied as a risk factor for malnutrition in in the elderly but it was not statistically significant so it was surprising that in our study it remained significant in the multiple regression analysis [28,29]. If this finding is confirmed by subsequent studies it might represent a valuable risk marker for malnutrition in surgical patients. In most of the studies which analyze variables associated with malnutrition in non-surgical elderly patients in a hospital or in the community, the measures of functional dependence, frailty and the indices of comorbidity dominate in the multiple regression analysis [27]. This was not the case in the present study, where the site of the operation was the central predictor of malnutrition risk because it represented diseases with detrimental effects on food intake and metabolism such as the gastrointestinal cancer. Moreover, the functional independence and the comorbidities of the patients are outweighed by ASA physical status which largely includes those elements by definition. Finally, it was not appropriate to use standardized frailty definitions in our analyses because in the frailty definitions that we have published in the past, malnutrition risk is a defining factor [30,31]. Instead, we used measures of functional capacity and comorbidity which do not include malnutrition indices.

The results of this study show an association of positive MUST screening with postoperative complications. Moreover, they suggest the MUST score as an independent predictor of hospital stay and postoperative death. These results are in agreement with the literature. Gn et al. reported that the MUST score ≥ 2 is an independent predictor of postoperative complications and length of stay in older patients undergoing elective general, gynecology and orthopedic surgery [20]. Among older patients undergoing gastrectomy for cancer in Japan, malnutrition risk assessed by the GNRI was an independent predictor of postoperative complications [9]. In studies not confined to the elderly, MUST score was an independent predictor of postoperative morbidity, mortality and length of stay in abdominal gastrointestinal surgery using the MUST and other nutrition screening tools [6,21,32]. The etiology of this association is multifactorial. Malnutrition affects the immune system, increases the risk for infections, the musculoskeletal system and impairs postoperative recovery, the respiratory function and it also has detrimental effects on wound healing [33]. Moreover it is associated with frailty, sarcopenia and increased comorbidities in the elderly which are dismal prognostic factors in surgery [30,34]. A growing body of evidence suggests that all these diverge consequences of malnutrition may share some common pathways. Given that the gut is the primary interface between dietary compounds and the immune system, it is expected that a range of cues from the microbiota, pathogens, and dietary components are required for healthy development of gut-associated lymphoid tissue (GALT). Indeed, various micronutrients and nutrient metabolites have been found to act as direct immune stimuli [35]. In addition to nutrient sensing, microbiota sensing via pathogen-recognition receptors (PRR) is also required for GALT development [36]. Defects in innate and adaptive immune function include for the former, impaired epithelial barrier function of the skin and gut, reduced granulocyte microbicidal activity, fewer circulating dendritic cells, and reduced complement proteins and for the later, reduced levels of soluble IgA in saliva and tears, lymphoid organ atrophy, reduced delayed-type hypersensitivity responses, fewer circulating B cells, a shift from Th1-associated to Th2-associated cytokines [37,38]. An example of the key pathway bridging nutritional status with immune regulation is the aryl hydrocarbon receptor (AhR). It is evidenced that intraepithelial lymphocytes abundantly express the AhR on their surface, which binds to metabolites of cruciferous vegetables. It is observed that intrinsic AhR signaling is essential for accurate lymphocyte localization in the gut and skin [39]. Moreover, lymphoid tissue-inducer cells express AhR and retinoic acid receptor (RAR)-related orphan receptor (ROR) γt, which interacts with the vitamin A metabolite retinoic acid, demonstrating a mechanistic link between nutrient sensing and immune development [40]. Given that proper function of the immune system plays a pivotal role for the surgical patient [41] proper nutrient supply is proved to positively correlate with key components of postoperative course. Indeed, immunonutrients (nutrients with measurable effect on the immune system through their supplementation) such as polyunsaturated/omega-3 fatty acids, arginine, glutamine, antioxidants, and nucleotides, have been found to reduce infection rates and the length of the hospital stay [41,42].

On the other hand, malnutrition is a partially modifiable risk factor for postoperative complications. As a matter of fact, preoperative nutritional conditioning which includes nutritional counselling, fortified diets, oral nutritional supplements and where necessary parenteral nutrition should be considered obligatory according to recent ESPEN guidelines, particularly in malnourished patients undergoing abdominal operations for malignancy [18,43]. There is evidence from a meta-analysis of studies that preoperative oral nutrition supplementation is linked with reduced mortality by 35%, reduced complications by 35% and reduced hospital stay by 2 days [44]. Furthermore, this practice was shown to be cost effective reducing the overall costs by a mean rate of 12.2% per patient in a meta-analysis of studies [44]. This is why nutritional conditioning is a pillar of prehabilitation programs which have gained popularity in recent times combining structured physical exercise, nutrition interventions, optimization of medical problems and even psychological support up to 6 weeks prior to major abdominal surgery. This strategy has the potential to significantly reduce the incidence of postoperative complications, to maintain the functional capacity of the patients in the postoperative period, to reduce the duration of postoperative stay and to improve the quality of life of the patients [7].

A limitation of this study is the non-homogeneous population in terms of surgical procedures, which were grouped by anatomical site and POSSUM magnitude creating two very powerful variables which dominated in the multiple regression models. This, along with the relatively small sample, was perhaps a reason why MUST did not retain its significance in the logistic regression model for predicting postoperative complications other than death. On the other hand, due to the fact that we did not exclude any group of older patients, we could show with pragmatic data that this screening method has the potential to predict outcomes in non-selected patients of a tertiary general surgery department. Moreover, since our study was retrospective we did not have the opportunity to assess malnutrition using more screening tools and biochemical or anthropometric variables. Instead, we used 17 variables which were relevant to malnutrition and postoperative outcomes in geriatric surgery which were prospectively registered with negligible rates of missing data. Most importantly we prospectively graded and recorded all postoperative complications including the minor ones, which were the endpoints.

## 5. Conclusions

This study showed a high prevalence of malnutrition risk in older patients undergoing general surgery which was associated with a history of smoking, gastrointestinal operations and worse physical status according to ASA classification. The MUST score was an independent predictor of postoperative length of stay and death. There results underscore the value for routine preoperative malnutrition screening in older patients with poor physical status undergoing gastrointestinal surgery in order to identify those who should benefit from perioperative nutritional conditioning.

## Figures and Tables

**Table 1:** Preoperative factors associated with malnutrition risk in older patients undergoing general surgery.

	MUST = 0 *n* (%)	MUST ≥ 1 *n* (%)	*p* *
Gender female	114 (40)	104 (48)	0.057
Age (65–74 yrs)	149 (53)	107 (50)	0.095
Education (0–12 yrs)	249 (90)	181 (88)	0.333
Living with partner	201 (72)	136 (65)	0.063
No of children Median (IQR)	2 (2)	2(1)	0.236 ^
Own house	261 (93)	201 (95)	0.440
Financial status (independent)	215 (79)	144 (70)	0.043
Smoking in the previous year	131 (47)	76 (36)	0.015
Alcohol intake	39 (14)	30 (14)	0.985
Katz ADL categories			
Dependent	14 (5)	24 (11)	0.020
Intermediate	22 (8)	20 (9)	
Independent	248 (87)	168 (79)	
History of falls	8 (3)	18 (9)	0.005
Charlson’s index			
0	100 (35)	45 (21)	0.000
1–2	123 (43)	81 (38)	
3–4	44 (15)	52 (24)	
>4	18 (6)	35 (16)	
Diagnosis of cancer	84 (30)	104 (49)	0.000
Diagnosis of dementia	16 (6)	24 (11)	0.020
POSSUM category			
Minor	43 (15)	14 (7)	0.000
Intermediate	111 (39)	44 (21)	
Major	103 (36)	108 (51)	
Major plus	27 (10)	47 (22)	
ASA class			
0–I	97 (35)	44 (21)	0.000
II	139 (50)	99 (47)	
III–IV	43 (15)	66 (32)	
Site of operation			
Hernia	83 (29)	25 (12)	0.000
Upper GI	4 (1)	18 (8)	
HPB	24 (8)	27 (13)	
Cholecystectomy	92 (32)	45 (21)	
Lower GI	48 (17)	86 (40)	
Soft tissue/thyroid/other	35 (12)	14 (7)	

MUST: malnutrition universal screening tool, OR (95% CI): odds ratio and its respective 95% confidence interval, ADL: activities of daily living, POSSUM: Physiological and Operative Severity Score for the enumeration of Mortality, ASA: American Society of Anesthesiologists. * Pearson’s chi square test, ^ Mann–Whitney test, Missing values < 3% for each variable.

**Table 2:** Multiple regression analysis of factors associated with malnutrition risk (MUST score ≥ 1) in older patients undergoing surgery.

	OR (95% CI)	*p* *
Smoking in the previous year	1.6 (1.01–2.5)	0.041
Surgical site		
Hernia	Ref.	0.000
Upper GI	20.4 (5.4–77.2)	0.000
HPB	3.7 (1.7–8.2)	0.001
Cholecystectom	1.1 (0.5–2.0)	0.866
Lower GI	5.2 (2.7–9.9)	0.000
Other ^	1.4 (0.7–2.5)	0.354
ASA class		
0/I	Ref.	0.003
II	1.7 (1.0–2.8)	0.053
III/IV	2.8 (1.5–5.2)	0.001

MUST: malnutrition universal screening tool, OR (95% CI): odds ratio and its respective 95% confidence interval, ASA: American Society of Anesthesiologists, * Wald test, ^ Soft tissue, breast, thyroid, Odds Ratios adjusted for diagnosis of cancer, dementia, Katz-ADL categories, dementia, Charlson’s comorbidity index, POSSUM category and financial status.

**Table 3:** Preoperative factors associated with postoperative complications in 501 older patients undergoing general surgery.

	Total *n* (%)	Any Compl. *n* (%)	*p* *	Serious Compl. *n* (%)	*p* *	Postop. Death *n* (%)	*p* *
MUST score							
0	286 (57)	66 (45)	0.001	15 (41)	0.079	2 (13)	0.001
1	142 (28)	49 (34)		16 (43)		8 (50)	
≥2	73 (15)	31 (21)		6 (16)		6 (37)	
Smoking in the previous year	207 (42)	59 (41)	0.743	17 (46)	0.586	5 (31)	0.389
Alcohol intake	69 (14)	26 (18)	0.076	10 (27)	0.019	2 (12)	0.853
Katz ADL categories							
Dependent	38 (8)	14 (10)	0.424	7 (19)	0.021	6 (40)	<0.001
Intermediate	42 (9)	10 (7)		2 (6)		0	
Independent	416 (84)	119 (83)		27 (75)		9 (60)	
History of falls	26 (5)	11 (8)	0.130	5 (14)	0.021	2 (13)	0.193
Polypharmacy (≥5)	161 (32)	49 (34)	0.690	14 (38)	0.457	5 (31)	0.925
Charlson’s index							
0	145 (29)	26 (20)	<0.001	7 (19)	0.024	3 (19)	0.001
1–2	204 (41)	59 (41)		11 (30)		2 (13)	
3–4	96 (19)	40 (28)		11 (30)		5 (31)	
>4	53 (11)	20 (14)		8 (22)		6 (38)	
Diagnosis of cancer	188 (38)	79 (55)	<0.001	20 (54)	0.039	8 (50)	0.322
Diagnosis of dementia	40 (8)	12 (9)	0.872	2 (5)	0.533	1 (6)	0.783
Emergency operation	85 (17%)	15 (22)	0.229	8 (21)	0.437	4 (25)	0.387
POSSUM category							
Minor	57 (12)	11 (8)	<0.001	4 (11)	0.012	0	0.084
Intermediate	155 (31)	23 (16)		3 (8)		2 (13)	
Major	211 (43)	75 (52)		23 (62)		11 (69)	
Major plus	74 (15)	35 (24)		7 (19)		3 (19)	
ASA class							
0–I	141 (29)	34 (24)	0.010	9 (24)	0.063	0	0.005
II	238 (49)	63 (45)		14 (38)		8 (50)	
III–IV	109 (22)	44 (31)		14 (38)		8 (50)	
Site of operation							
Hernia	108 (22)	16 (11)	<0.001	5 (14)	0.016	1 (6)	0.042
Upper GI	22 (4)	9 (6)		1 (3)		1 (6)	
HPB	51 (10)	30 (21)		8 (22)		3 (19)	
Cholecystectomy	137 (27)	29 (20)		8 (22)		2 (13)	
Lower GI	134 (27)	54 (37)		15 (22)		9 (56)	
Soft tissue/thyroid/other	49 (10)	8 (6)		0		0	

MUST: malnutrition universal screening tool, ADL: activities of daily living, POSSUM: Physiological and Operative Severity Score for the enumeration of Mortality, ASA: American Society of Anesthesiologists, * Pearson’s chi square, Missing values < 3% for each variable.

**Table 4:** Univariate analysis odds ratio (OR) and multiple regression analysis adjusted OR (AOR) for postoperative complications and postoperative length of stay.

	Any Complication			
MUST Score	OR (95% CI)	*p*	AOR (95% CI)	*p*
Low (0)	Ref.		Ref. *	
Medium (1)	1.8 (1.2–2.8)	0.009	1.1 (0.7–1.9)	0.720
High (≥2)	2.0 (1.2–3.4)	0.007	1.3 (0.7–2.6)	0.409
	Serious complication (Clavien–Dindo ≥ III)			
Low (0)	Ref.		Ref. ^	
Medium (1)	2.3 (1.1–4.8)	0.027	1.2 (0.7–2.1)	0.438
High (≥2)	1.4 (0.5–3.8)	0.494	1.4 (0.7–2.6)	0.337
	Postoperative death			
Low (0)	Ref.		Ref. **	
Medium (1)	8.5 (1.8–40.5)	0.007	9.1 (1.1–80.3)	0.047
High (≥2)	11.1 (2.2–56)	0.004	11.9 (1.2–121)	0.035
	Postoperative stay			
Low (0)	3(7) ^#^	<0.001 ^##^	Ref. ^^	
Medium (1)	9(10)		1.3 (1.01–1.6)	0.041
High (≥2)	9(10)		1.7 (1.5–2.0)	<0.001

MUST: malnutrition universal screening tool, OR: odds ratio, AOR: adjusted odds ratio, 95% CI: 95% confidence interval, ASA: American Society of Anesthesiologists, ADL: activities of daily living, POSSUM: Physiological and Operative Severity Score for the enumeration of Mortality, * Adjusted for, Site of operation, POSSUM category, Diagnosis of cancer, Charlson’s index, Recent admission, Katz ADL, ASA. ^ Adjusted for Site of operation, POSSUM category, Diagnosis of cancer, Charlson’s index, Katz ADL, ASA. ** Adjusted for Site of operation, Charlson’s index, Katz ADL, ASA. ^^ Negative binomial regression adjusted incidence rate ratio. Adjusted for site of the operation, Charlson’s index, ASA class, polypharmacy and diagnosis of cancer. ^#^ Days, Median (interquartile range), ^##^ Mann–Whitney U test.

## Data Availability

All data generated or analyzed during this study are included in this published article or are available from the corresponding author upon reasonable request.

## Supplementary Materials

The following are available online at https://www.mdpi.com/article/10.3390/jcm10122550/s1. Table S1. Preoperative factors associated with malnutrition risk in older patients undergoing general surgery. Table S2. Multivariate logistic regression analysis of factors associated with postoperative death. Table S3. Multivariate logistic regression analysis of factors associated with serious postoperative complications. Table S4. Multivariate logistic regression analysis of factors associated with any postoperative complications. Table S5. Multivariate negative binomial regression analysis of factors associated with postoperative stay.
