# Supplementary material for: Factors Associated with Malnutrition and Its Impact on Postoperative Outcomes in Older Patients
Source: J Clin Med. 2021 Jun 9;10(12):2550. doi: 10.3390/jcm10122550 (PMC8229217; doi:10.3390/jcm10122550)
Supplement: Supplementary file 1 [file jcm-10-02550-s001.zip › jcm-1191577-supplementary.pdf]

Journal of
Clinical Medicine

MDPI

Supplementary Materials

Supplementary Table S1. Preoperative factors associated with malnutrition risk in older patients undergoing general surgery.

	MUST = 0 *n* (%)	MUST = 1 *n* (%)	MUST > 1 *n* (%)	*p**
**Female gender**	114 (40)	67 (47)	37 (51)	0.145
**Age groups**				
65–69yrs	86 (30)	34 (24)	17 (23)	0.324
70–74yrs	65 (23)	37 (26)	19 (26)	
75–79yrs	77 (27)	33 (24)	15 (21)	
>79rys	56 (20)	36 (26)	22 (30)	
**Educations (yrs)**				
0–6	169 (61)	88 (65)	40 (56)	0.263
6–12	80 (29)	29 (22)	24 (34)	
>12	29 (10)	18 (13)	7 (10)	
**Living with partner**	201 (72)	90 (65)	46 (63)	0.169
**No of children Median(IQR)**	2 (2)	2 (1)	2 (1)	0.000^
**Own house**	261 (93)	132 (96)	69 (95)	0.601
**Financial status (independent)**	215 (79)	84 (64)	60 (82)	0.002
**Smoking in the previous year**	131 (47)	49 (35)	27 (37)	0.053
**Alcohol consumption**	39 (14)	21 (15)	9 (12)	0.877
**Diagnosis of cancer**	84 (30)	59 (42)	45 (63)	0.000
**Diagnosis of dementia**	16 (6)	19 (14)	5 (7)	0.018
**Charlson's index**				
0	100 (35)	33 (23)	12 (17)	0.000
1–2	123 (43)	55 (39)	26 (36)	
3–4	44 (15)	33 (23)	19 (26)	
5+	18 (6.3)	20 (14)	15 (21)	
**History of falls**	8 (3)	13 (9)	5 (7)	0.017
**POSSUM category**				
minor	43 (15)	12 (9)	2 (3)	0.000
intermediate	111 (39)	32 (23)	12 (16)	
major	103 (36)	68 (49)	40 (55)	
major plus	27 (10)	28 (20)	19 (26)	
**ASA class**				
0/I	97 (35)	30 (21)	14 (20)	0.000
II	139 (50)	72 (51)	27 (39)	
III/IV	43 (15)	38 (27)	28 (41)	
**Site of operation**				
hernia	83 (29)	22 (16)	3 (4)	0.000
upper GI	4 (1)	12 (9)	6 (8)	
HPB	24 (8)	18 (13)	9 (12)	
cholecystectomy	92 (32)	35 (25)	10 (14)	
lower GI	48 (17)	50 (35)	36 (49)	
breast/soft tissue/thyroid/other	35 (12)	5 (4)	9 (12)	
**Katz ADL categories**				
Dependent	14 (5)	10 (7)	14 (19)	0.000
Intermediate	22 (8)	10 (7)	10 (14)	

Independent	248 (87)	120 (86)	48 (67)

MUST: malnutrition universal screening tool, OR (95%CI): odds ratio and its respective 95% confidence interval, ADL: activities of daily living, POSSUM: Physiological and Operative Severity Score for the enUmeration of Mortality, ASA: American Society of Anaesthetists *Pearson's chi square test, ^Mann-Whitney test, Missing values <3% for each variable.

Supplementary Table S2. Multivariate logistic regression analysis of factors associated with postoperative death.

	B	S.E.	Wald	df	p*	OR	95% C.I. for OR	
							Lower	Upper
**MUST score**								
0			6.9	2	0.0			
1	3.1	1.2	6.4	1	0.04	9.1	1.1	80.3
≥2	3.3	1.3	6.5	1	0.03	11.9	1.2	121
**Charlson's index**								
0			5.2	3	0.16			
1–2	-1.7	1.1	2.3	1	0.13	0.2	0.02	1.6
3–4	-0.9	0.9	0.9	1	0.33	0.4	0.05	2.7
>4	0.1	1.0	0.02	1	0.99	1.1	0.2	8.8
**Surgical site**								
Hernia			1.4	5	0.93			
Upper GI	-0.4	1.7	0.05	1	0.83	0.7	0.02	18.1
HPB	0.6	1.4	0.2	1	0.66	1.9	0.1	29.0
Lap. Chole.	-0.8	1.4	0.3	1	0.57	0.5	0.03	7.1
Lower GI	0.3	1.3	0.04	1	0.84	1.3	0.1	14.9
Other	-17.7	4905.2	0.00	1	0.99	0.0	0.0	.
**ASA class**								
ASA 0/I			0.2	2	0.89			
ASA II	17.2	2961	0.00	1	0.99	.	0.0	.
ASA III/IV	17.2	2961	0.00	1	0.99	.	0.0	.
**Katz ADL categories**								
Dependent			7.2	2	0.03			
Intermediate	-19.3	5385.3	0.00	1	0.99	0.0	0.0	.
Independent	-2.2	0.8	7.2	1	0.01	0.1	0.02	0.5
**Emergency operation**	1.6	0.9	3.1	1	0.08	4.7	0.8	26.6
**Diagnosis of Dementia**	1.1	1.2	0.7	1	0.39	2.9	0.3	31.7
**Constant**	-23.3	2961.9	0.00	1	0.99	0.0		

* Wald test significance. S.E.: standard error, Df: degrees of freedom, OR: Odds Ratio, 95% C.I.: 95% confidence interval, MUST: malnutrition universal screening tool, Upper GI: upper gastrointestinal tract, HPB: hepatopancreatobilliary, Lap Chole: laparoscopic cholecystectomy, ASA: American Society of Anaesthetists, ADL: Activities of Daily Life.

Supplementary Table S3. Multivariate logistic regression analysis of factors associated with serious postoperative complications.

	B	S.E.	Wald	df	p*	OR	95% C.I. for OR	
							**Lower**	**Upper**
**MUST score**								
0			2.1	2	0.36			
1	0.6	0.5	1.9	1	0.44	1.2	0.7	2.1
≥2	0.3	0.6	0.2	1	0.34	1.4	0.7	2.6
**Charlson's index**								
0			1.2	3	0.75			
1–2	-0.2	0.6	0.1	1	0.71	0.8	0.3	2.5
3–4	0.1	0.7	0.02	1	0.89	1.1	0.3	3.9

>4	0.4	0.8	0.3	1	0.57	1.5	0.4	6.9
**Surgical site**								
Hernia			4.9	5	0.43			
Upper GI	-0.3	1.4	0.03	1	0.86	0.8	0.05	12.8
HPB	1.4	1.0	1.8	1	0.17	4.1	0.5	31.1
Lap. Cholecystect.	0.9	0.9	0.9	1	0.34	2.5	0.4	16.1
Lower GI	0.4	0.9	0.2	1	0.66	1.5	0.2	9.7
Other	-18.3	6061	0.00	1	0.99	0.0	0.00	.
**POSSUM category**								
Minor			10.1	3	0.02			
Intermediate	-2.4	1.1	4.4	1	0.04	0.1	0.01	0.9
Major	-0.3	0.9	0.1	1	0.75	0.7	0.1	5.1
Major plus	-1.1	1.2	0.9	1	0.33	0.3	0.03	3.1
**Katz ADL categories**								
Very dependent			7.2	2	0.03			
Intermediate	-1.8	0.9	3.9	1	0.04	0.2	0.02	0.9
Independent	-1.6	0.6	6.7	1	0.00	0.2	0.06	0.7
**Alcohol consumption**	-0.9	0.5	4.3	1	0.03	0.4	0.2	0.9
**ASA class**								
ASA 0/I			1.6	2	0.45			
ASA II	-0.4	0.5	0.5	1	0.48	0.7	0.3	1.8
ASA III/IV	0.2	0.6	0.2	1	0.68	1.3	0.4	3.7
**Diagnosis of cancer**	-0.2	0.5	0.1	1	0.75	0.9	0.3	2.4
**Emergency operation**	-0.8	0.6	1.8	1	0.17	0.5	0.2	1.4
Constant	0.9	1.3	0.5	1	0.46	2.6		

*Wald test significance. S.E.: standard error, Df: degrees of freedom, OR: Odds Ratio, 95% C.I.: 95% confidence interval, MUST: malnutrition universal screening tool, Upper GI: upper gastrointestinal tract, HPB: hepatopancreatobilliary, Lap Chole: laparoscopic cholecystectomy, ASA: American Society of Anaesthetists, ADL: Activities of Daily Life.

Supplementary Table S4. Multivariate logistic regression analysis of factors associated with any postoperative complications.

	**B**	**S.E.**	**Wald**	**df**	***p****	**OR**	**95% C.I. for OR**	
							Lower	Upper
**MUST score**								
0			2.9	2	0.23			
1	0.4	0.3	1.7	1	0.72	1.1	0.7	1.9
≥2	0.5	0.3	2.3	1	0.41	1.3	0.7	2.6
**Charlson's index**								
0			0.7	3	0.86			
1–2	0.1	0.3	0.1	1	0.79	1.1	0.5	2.0
3–4	0.2	0.4	0.3	1	0.56	1.3	0.5	2.6
>4	-0.1	0.5	0.02	1	0.88	0.9	0.3	2.3
**Diagnosis of cancer**	-0.5	0.3	2.1	1	0.15	0.6	0.3	1.1
**Surgical site**								
Hernia			16.0	5	0.01			
Upper GI	1.2	0.6	3.0	1	0.08	3.2	0.8	11.9
HPB	1.9	0.6	10.5	1	0.00	6.8	2.1	22.0
Lap. Cholecystect.	0.9	0.5	3.3	1	0.07	2.3	0.9	6.0
Lower GI	0.9	0.5	3.7	1	0.05	2.5	0.9	6.7
Other	-0.1	0.5	0.1	1	0.81	0.9	0.2	2.7

**POSSUM category**								
Minor			7.8	3	0.05			
Intermediate	-1.1	0.5	4.1	1	0.04	0.3	0.1	0.9
Major	-0.2	0.5	0.2	1	0.64	0.8	0.2	2.1
Major plus	-0.6	0.6	1.0	1	0.31	0.5	0.1	1.7
**Katz ADL categories**								
Very dependent			1.2	2	0.53			
Intermediate	-0.6	0.6	1.2	1	0.26	0.5	0.1	1.6
Independent	-0.3	0.4	0.5	1	0.44	0.7	0.3	1.6
**ASA class**								
ASA 0/I			3.5	2	0.17			
ASA II	-0.1	0.3	0.1	1	0.74	0.9	0.5	1.5
ASA III/IV	0.4	0.3	1.7	1	0.18	1.5	0.8	2.9
**Emergency operation**	-0.6	0.3	3.0	1	0.08	0.5	0.2	1.1
Constant	-0.9	0.7	1.6	1	0.20	0.4		

*Wald test significance. S.E.: standard error, Df: degrees of freedom, OR: Odds Ratio, 95% C.I.: 95% confidence interval, MUST: malnutrition universal screening tool, Upper GI: upper gastrointestinal tract, HPB: hepatopancreatobilliary, Lap Chole: laparoscopic cholecystectomy, ASA: American Society of Anaesthetists, ADL: Activities of Daily Life.

Supplementary Table S5. Multivariate negative binomial regression analysis of factors associated with postoperative stay.

Parameter	B	S.E.	95% Wald CI		Hypothesis Test			IRR	95%CI for IRR	
			Lower	Upper	Wald χ^2	df	p*		Lower	Upper
(Intercept)	1.3	0.2	0.8	1.8	29.0	1	0.00	3.7	2.3	6.1
**MUST score**										
0	0	.	.	.	.	.	.	1	.	
1	0.4	0.1	0.2	0.6	20.6	1	0.04	1.3	1.01	1.8
≥2	0.1	0.1	-0.1	0.3	1.1	1	0.00	1.7	1.5	2.0
**Age groups**										
65–69	0	.	.	.	.	.	.	1	.	.
70–74	0.1	0.1	-0.1	0.3	1.6	1	0.19	1.1	0.9	1.3
75–79	-0.1	0.1	-0.3	0.1	0.4	1	0.50	0.9	0.7	1.1
+80	-0.04	0.1	-0.3	0.1	0.1	1	0.70	0.9	0.7	1.1
**Charlson's index**										
0	0	.	.	.	.	.	.	1	.	.
1–2	-0.04	0.1	-0.2	0.2	0.1	1	0.66	0.9	0.7	1.1
3–4	-0.04	0.1	-0.3	0.2	0.1	1	0.79	0.9	0.7	1.2
>4	0.02	0.1	-0.3	0.3	0.01	1	0.90	1.0	0.7	1.3
**Diagnosis of cancer**										
Yes	0	.	.	.	.	.	.	1	.	.
No	-0.1	0.1	-0.3	0.1	1.01	1	0.31	0.9	0.7	1.1
**Surgical site**										
Hernia	0	.	.	.	.	.	.	1	.	.
Upper GI	1.1	0.2	0.8	1.5	36.4	1	0.00	3.2	2.1	4.6
HPB	1.4	0.2	1.1	1.7	90.1	1	0.00	4.1	3.0	5.5
Lap. Cholecystect.	0.05	0.1	-0.2	0.3	0.1	1	0.66	1.1	0.8	1.2
Lower GI	0.8	0.1	0.6	1.1	45.6	1	0.00	2.3	1.8	2.9
Other	0.4	0.1	0.1	0.7	7.9	1	0.01	1.5	1.1	2.0

**ASA class**										
ASA 0/I	0	.	.	.	.	.		1	.	.
ASA II	0.1	0.1	-0.1	0.3	1.3	1	0.24	1.1	0.9	1.3
ASA III/IV	0.2	0.1	0.1	0.5	6.9	1	0.01	1.3	1.0	1.6
**Polypharmacy**										
Yes	0	.	.	.	.	.	.	1	.	.
No	-0.1	0.1	-0.3	0.01	3.1	1	0.08	0.8	0.7	1.0
**History of falls**										
Yes	0	.	.	.	.	.	.	1	.	.
No	0.1	0.2	-0.2	0.4	0.2	1	0.68	1.1	0.8	1.4
**Emergency operation**										
No	0	.	.	.	.	.	.	1	.	.
Yes	0.3	0.1	0.1	0.5	5.7	1	0.02	1.3	1.1	1.6

*Wald test significance. S.E.: standard error, df: degrees of freedom, IRR: Incidence Rate Ratio, 95% C.I.: 95% confidence interval, MUST: malnutrition universal screening tool, Upper GI: upper gastrointestinal tract, HPB: hepatopancreatobilliary, Lap Chole: laparoscopic cholecystectomy, ASA: American Society of Anaesthetists, ADL: Activities of Daily Life
